# Impact of local air quality management policies on emergency hospitalisations for respiratory conditions in the North West Coast region of England: a longitudinal controlled ecological study

**DOI:** 10.1186/s12939-021-01598-w

**Published:** 2021-12-13

**Authors:** Tanith C. Rose, Konstantinos Daras, Jane Cloke, Sarah Rodgers, Paul Farrell, Saiqa Ahmed, Benjamin Barr

**Affiliations:** 1 Institute of Population Health Sciences, University of Liverpool, L69 3GL Liverpool, UK; 2 Environmental Protection & Public Protection Enforcement, Liverpool City Council, Liverpool, UK; 3 Public Advisor NIHR Applied Research Collaboration North West Coast, Liverpool, UK

**Keywords:** Air pollution, Policy, Socioeconomic factors, Respiratory tract diseases

## Abstract

**Background:**

Air quality is monitored at a local level in the UK as part of the Local Air Quality Management (LAQM) system. If air quality objectives within an area are not achieved an Air Quality Management Area (AQMA) is declared and action plan developed. The efficacy of this system in reducing air pollution has increasingly come into question, however very little is known about its impact on health or health inequalities. We therefore investigated the effect of declaring an AQMA on emergency hospitalisations for respiratory conditions in the North West Coast region of England, and examined whether the effect differed between more compared to less deprived neighbourhoods.

**Methods:**

This longitudinal controlled ecological study analysed neighbourhoods located within or touching the boundaries of AQMAs declared in the North West Coast region between 2006 and 2016. Each of these intervention neighbourhoods were matched with five control neighbourhoods which had never been located within/touching an AQMA boundary. Difference-in-differences methods were used to compare the change in hospitalisation rates in the intervention neighbourhoods to the change in hospitalisation rates in the matched control neighbourhoods, before and after the declaration of an AQMA.

**Results:**

In total, 108 intervention neighbourhoods and 540 control neighbourhoods were analysed over the period 2005–2017, giving a total sample size of 8424 neighbourhood-years. Emergency hospitalisations for respiratory conditions decreased in the intervention neighbourhoods by 158 per 100,000 per year [95% CI 90 to 227] after an AQMA was declared relative to the control neighbourhoods. There was a larger decrease in hospitalisation rates following the declaration of an AQMA in more compared to less income deprived neighbourhoods.

**Conclusions:**

Our results suggest the LAQM system has contributed to a reduction in emergency hospitalisations for respiratory conditions, and may represent an effective strategy to reduce inequalities in health. These findings highlight the importance of measuring the success of air quality policies not just in terms of air pollution but also in terms of population health.

**Supplementary Information:**

The online version contains supplementary material available at 10.1186/s12939-021-01598-w.

## Background

Air pollution poses a substantial threat to health, contributing to an estimated seven million premature deaths worldwide every year [1]. Exposure to air pollution has important short- and long-term health consequences, and can have detrimental effects across the life course. Short-term exposure to elevated levels of air pollution has been linked to a range of health effects, including decreased lung function, exacerbation of asthma symptoms, and increases in respiratory and cardiovascular hospital admissions [2–4]. Long-term exposure to air pollution has been associated with the development and progression of various cardiovascular and respiratory diseases, diabetes and mortality from cancer [5–7]. Public Health England estimate that the costs due to the health impacts of air pollution to the National Health Service (NHS) and social care system alone could reach £5.3 billion by 2035 in England [8].

Since air pollution is worse in more deprived communities, it is a major cause of health inequalities. Socioeconomically disadvantaged individuals are more likely to be exposed to higher levels of air pollution in the home, work, at school and when commuting [9]. Additionally, air pollution can exacerbate pre-existing cardiovascular and respiratory conditions, and these conditions tend to be more prevalent and more severe amongst lower socioeconomic groups. The enduring disproportionate impact of air pollution on deprived communities has been described as a clear environmental injustice, given that air quality standards are intended to protect everyone [10].

Following the Environment Act 1995, the UK government introduced a system of Local Air Quality Management (LAQM) to tackle air pollution, overseen by the Department for Environment, Food and Rural Affairs (Defra) in England [11]. Local authorities are required to regularly assess local air quality, and are expected to report on concentrations of nitrogen dioxide (NO_2_), particulate matter < 10 μm and sulphur dioxide on an annual basis [12]. If national air quality objectives within an area are not being achieved or are not likely to be achieved, local authorities are required to designate an Air Quality Management Area (AQMA) [12]. Once an AQMA has been designated, an action plan detailing the measures that will be taken to tackle the problem should ideally be prepared within a year [12]. Action plan measures are tailored to best address the source of the problem in each area, but may include traffic management to reduce congestion, cycling/walking infrastructure improvements, and public transport improvements. A summary of measures included in action plans from local authorities in the North West Coast region are provided in Supplementary file, Appendix 1.

Key strengths of the LAQM system include the ability to identify small areas experiencing poor air quality which would otherwise be overlooked at a national level, and to utilise local knowledge to implement context appropriate solutions [13]. However, the efficacy of the LAQM system in reducing air pollution has increasingly come into question [14]. Since 2001, around a third of AQMAs in England have been revoked following sustained improvements in air quality [15]. Even though local authorities are encouraged to consider the potential benefits of not revoking successful AQMAs [16], these figures have prompted suggestions that the current framework has not produced sufficient action at a local level [15]. At a national level, there is little evidence to suggest air quality has improved since the LAQM system was introduced in 1998 [13]. Whether these criticisms reflect failure of national air quality policies as opposed to local measures has been debated [17], however a major barrier to assessing the effectiveness of the LAQM system is the lack of evaluation evidence utilising robust controlled methods.

Evaluating the effects of local action plan measures can, and has, proved challenging [18], not least because up-to-date information on action plan measures are not routinely provided by Defra. Defra does however provide data on AQMA locations and declaration dates. Since AQMAs are introduced in different areas at different time points, this natural variation can be utilised to evaluate differences in outcomes over time between AQMA areas and non-AQMA (control) areas using quasi-experimental methods. We identified one non-peer reviewed study which used these methods to compare changes in NO_2_ concentrations between local authorities that had ever declared an AQMA to those that had never declared an AQMA, and found no evidence that active AQMAs were associated with reductions in NO_2_ concentrations [19]. However, we were unable to identify any previous studies utilising similar methods at a small-area level, or which examined outcomes other than air pollution, such as health or inequalities in health.

National air quality objectives are based on minimising the risk to human health, however the effects of local measures on health outcomes are not routinely evaluated [13] [20]. In general, evaluation evidence of the impact of air quality interventions on health outcomes is lacking [21]. Furthermore, despite research which suggests environmental inequalities in the UK have increased over time [10], very little is known about the effect of air quality strategies in general on health inequalities [9]. Studies which utilise robust methods are needed to move beyond describing environmental injustices to identifying solutions to tackle the issue [22]. To address these substantial gaps in the literature, we investigated the impact of declaring an AQMA on emergency admission rates for respiratory conditions in the North West Coast region of England, and examined whether the impact was different in more deprived compared to less deprived communities.

## Methods

### Air quality management areas in the north west coast

We analysed data from the North West Coast region of England which covers 29 local authorities. In total, 78 AQMAs have been declared in the North West Coast (see Supplementary file, Appendix 2 for details). Of these 48 were declared within our intervention time frame between 2006 and 2016. This time frame corresponded with data availability of the outcome measure, permitting pre/post-AQMA declaration measurements to be taken. Since the AQMAs were declared at different points in time within the intervention time frame, the number of years of pre/post measurements varied by AQMA. One city-wide AQMA declared in 2009 by Liverpool City Council coincided with the introduction of a primary care incentive scheme in Liverpool, which aims included reducing emergency admissions for conditions such as asthma and chronic obstructive pulmonary disease (COPD). To limit potential confounding, this large AQMA which covered the city of Liverpool was excluded from the analysis.

The vast majority of the remaining 47 AQMAs were declared due to exceedences of NO_2_ concentrations, and eight have been revoked following air quality improvements (see Supplementary file). These eight AQMAs were analysed in exactly the same manner as the non-revoked AQMAs. This ‘Intention-to-treat’ approach provides a more accurate estimate of the intervention effect and is less prone to bias.

### Study design

We conducted a longitudinal matched controlled ecological study using difference-in-differences methods. Lower-layer Super Output Area (LSOA) -years were the units of analysis. LSOAs are small geographical areas used by the UK’s Office for National Statistics (ONS), each containing a population of between 1000 and 3000 people [23]. Within the text of this article, LSOAs will subsequently be referred to as neighbourhoods.

**Fig. 1:**
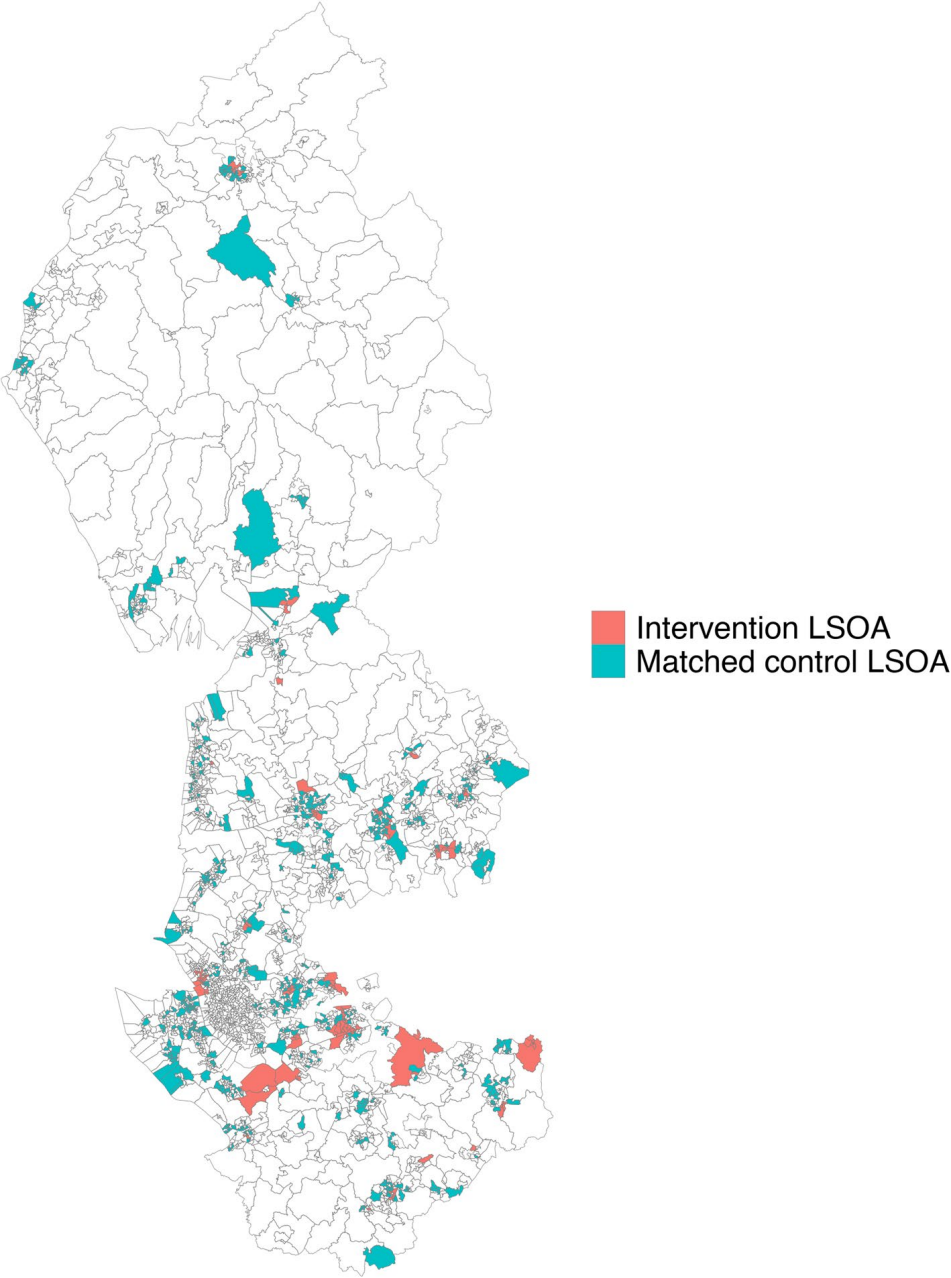
Intervention neighbourhoods and matched control neighbourhoods located within the North West Coast region of England

Included in the analysis were 108 neighbourhoods which were located within or touching/intersecting the boundaries of 47 AQMAs declared between 2006 and 2016 in the North West Coast. Each of these 108 intervention neighbourhoods were matched with five control neighbourhoods located within other areas within the North West Coast region which had never been located within or touching an AQMA boundary (see Fig. 1 for map of intervention and control neighbourhoods).

We then compared the change (difference) in outcomes in the intervention neighbourhoods to the change (difference) in outcomes in the matched control neighbourhoods, before and after the declaration of an AQMA. This difference-in-differences method controls for measured and un-measured time-invariant differences between the intervention and control neighbourhoods, as well as time-varying factors that affect the outcome in the same way between the neighbourhoods [24]. The key assumption of difference-in-differences analysis is the parallel trends assumption. If the trend in the outcome in the intervention and control neighbourhoods would have been parallel in the absence of the intervention then, the difference between the change in the outcomes between the two groups provides an unbiased estimate of the interventions effect [25]. This assumption becomes more plausible if the intervention and control neighbourhoods are similar to each other in terms of trends in the outcome in the pre-intervention period. We therefore investigated this assumption by examining trends between the intervention and control neighbourhoods prior to the intervention (see below and Supplementary file, Appendix 3 for further details).

### Data sources and measures

We obtained current and historical data from Defra on the geographical location of each AQMA via a Freedom of Information request. Location data were not available for one AQMA (Cheshire East, Congleton AQMA No.3) which was declared in 2005 and revoked in 2006. We used aggregated Hospital Episode Statistics (obtained via data sharing agreement with NHS Digital) on respiratory admissions per neighbourhood of residence and ONS population estimates [26] to derive our outcome for each neighbourhood: emergency hospital admissions for respiratory conditions per 100,000 population. Respiratory conditions included acute upper respiratory infections, influenza, pneumonia, acute and chronic bronchitis, emphysema, COPD and asthma (ICD-10 codes in Supplementary file, Appendix 4). To adjust for time varying factors that could be associated with trends in emergency admission rates for respiratory conditions we controlled for the annual percent of the population aged < 15 years and 65+ years, and the percent unemployed using data obtained from the ONS [26, 27].

Matching of the control to the intervention neighbourhoods was based on a range of socio-demographic characteristics including population size [26], income deprivation [28], urban/rural classification [29], asthma and COPD prevalence rates [30, 31], emergency admission rates for respiratory conditions, travelling distance to the nearest general practice and travelling distance to the nearest hospital with an Accident and Emergency department [32]. A full description of the measures and data sources can be found in Supplementary file, Appendix 4.

### Statistical analysis

We used propensity score matching to ensure that the control neighbourhoods had similar observed characteristics to the intervention neighbourhoods in the time period before the declaration of an AQMA. To perform the matching, we grouped the intervention neighbourhoods by their corresponding AQMA declaration year, and matched controls to the intervention neighbourhoods based on data from the time period before the declaration year. The nearest neighbour method was used for matching, which selects controls with propensity scores that are closest to that of the intervention subjects [33]. The reasons for matching were to identify control neighbourhoods that were likely to follow similar trends in the outcomes over time to the intervention neighbourhoods, as the difference-in-differences method controls for all fixed differences between the intervention and control neighbourhoods. In other words, the primary aim of using propensity score matching in our analysis was to help meet the parallel trend assumption, rather than to remove all differences in neighbourhood characteristics at baseline.

To estimate the difference-in-differences – i.e. the difference between the change in outcomes before and after the declaration of an AQMA in the intervention neighbourhoods, and the change in outcomes over the same time periods in the control neighbourhoods, we included an intervention group by time period interaction term in a linear regression model. To control for potential demographic and socioeconomic changes which may confound the result we included annual data on the percent of the population aged < 15 years and 65+ years, and the percent unemployed. We included an annual time trend term and a random intercept for each neighbourhood to account for the longitudinal nature of the data (see Supplementary file, Appendix 3 for full details of the statistical model). We performed subgroup analysis to investigate whether there were differential effects by income deprivation. Standard errors and confidence intervals were calculated using cluster robust estimation. Analyses were conducted using R (version 4.0.5).

### Robustness tests

The parallel trends assumption was tested using graphical methods and regression models to compare trends in the outcome of interest between the intervention and control neighbourhoods in the time period before the declaration of an AQMA. We also repeated the analysis including the Liverpool City Council city-wide AQMA to examine how this impacted the results.

## Results

In total, 108 intervention neighbourhoods and 540 control neighbourhoods from the North West Coast were analysed over the period 2005–2017, giving a total sample size of 8424 neighbourhood-years. Baseline characteristics of the neighbourhoods are shown in Table 1. Overall, the intervention neighbourhoods had similar baseline characteristics to the control neighbourhoods and the difference-in-differences analysis controls for any fixed differences between the groups.

**Table 1:** Baseline characteristics of the intervention and control neighbourhoods, averaged over the time period before AQMA declaration

	Intervention neighbourhoods Number = 108	Control neighbourhoods Number = 540
	Mean (SD)	Mean (SD)
Income deprivation score (%)	18.76 (11.06)	18.35 (12.91)
Distance to general practice (km)	1.02 (0.82)	1.05 (0.80)
Distance to hospital with A&E (km)	6.58 (4.24)	6.61 (5.59)
Prevalence of asthma (%)	6.25 (0.64)	6.33 (0.65)
Prevalence of COPD (%)	1.98 (0.54)	2.06 (0.54)
Population (number)	1588.35 (275.59)	1542.20 (264.53)
Annual emergency admission rate for respiratory conditions (per 100,000 population)	1302.27 (599.37)	1320.33 (644.73)
Percent classified as urban	94.1	94.4

*A&E* Accident and Emergency department; *COPD* Chronic obstructive pulmonary disease; *km* kilometres; *SD* standard deviation

**Fig. 2:**
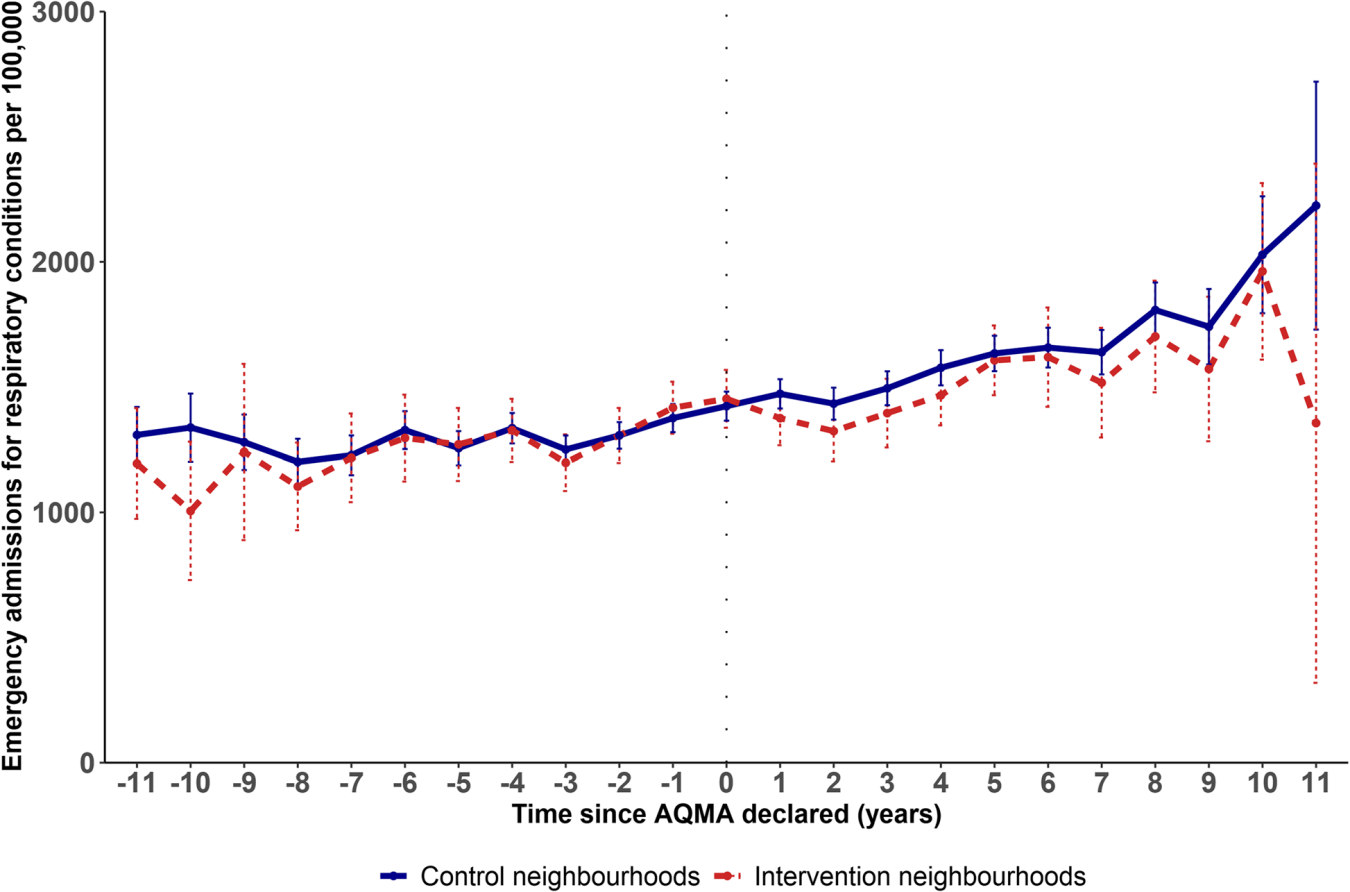
Trends in emergency admission rates for respiratory conditions for the intervention and control neighbourhoods, pre- and post-AQMA declaration

Figure 2 shows the trends in emergency admission rates for respiratory conditions for the intervention and control neighbourhoods, pre- and post-AQMA declaration. In the pre-intervention period, emergency admission rates were similar in the intervention and control neighbourhoods, and the trends appeared to be parallel – admission rates were generally increasing for both groups. Following the introduction of an AQMA, admission rates for the control neighbourhoods continued to increase, but rates for the intervention neighbourhoods decreased to levels lower than the rates observed in the control neighbourhoods, before increasing again.

Results from the difference-in-differences analysis for emergency admission rates for respiratory conditions are shown in Table 2. The coefficient for the difference-in-differences estimator indicates that on average emergency admissions for respiratory conditions decreased in the intervention neighbourhoods by 158 per 100,000 per year [95% CI 90 to 227] after an AQMA was declared relative to the control neighbourhoods. In relative terms, this is approximately equivalent to an annual 12% reduction in emergency admissions for respiratory conditions.

**Table 2:** Results of difference-in-differences analysis showing the change in emergency admissions for respiratory conditions per 100,000 population in the intervention neighbourhoods following the declaration of an AQMA relative to the control neighbourhoods

	Coefficient	95% CI	*p*-value
Working age population unemployed (%)	− 1.1	[− 10.21, 8.01]	0.812
Population aged < 15 years (%)	45.24	[36.76, 53.71]	< .001
Population aged 65+ years (%)	13.61	[7.20, 20.03]	< .001
Years to/since intervention	45.48	[40.04, 50.92]	< .001
Period [post-intervention = 1; pre-intervention = 0]	23.32	[−17.67, 64.32]	0.265
Group [intervention = 1; control = 0]	82.27	[−19.79, 184.34]	0.114
DiD estimator: Period * Group	−158.45	[−227.29, − 89.61]	< .001

Model based on equation shown in Supplementary file and includes random intercept for neighbourhood.

Model based on 108 intervention and 540 control neighbourhoods, and 8424 observations.

*CI* confidence interval; *DiD* Difference-in-Differences

The results shown in Table 3 indicate that there are statistically significant differential effects of declaring an AQMA across deprivation subgroups. In the least deprived subgroup, we found no statistically significant effect of declaring an AQMA. However, in the middle deprivation subgroup emergency admissions for respiratory conditions decreased in the intervention neighbourhoods by 184 per 100,000 per year [95% CI 68 to 301] after an AQMA was declared relative to the control neighbourhoods, and in the most deprived subgroup admissions decreased by 200 per 100,000 per year [95% CI 95 to 304].

**Table 3:** Results of difference-in-differences analysis showing the change in emergency admissions for respiratory conditions per 100,000 population in the intervention neighbourhoods following the declaration of an AQMA relative to the control neighbourhoods, stratified by income deprivation

	Model 1: Least deprived neighbourhoods		Model 2: Middle deprivation		Model 3: Most deprived neighbourhoods	
	Coefficient	95% CI	Coefficient	95% CI	Coefficient	95% CI
Working age population unemployed (%)	−12.7	[−44.35, 18.95]	−35.7	[− 52.97, −18.43]	−8.02	[−19.91, 3.87]
Population aged < 15 years (%)	15.84	[0.01, 31.67]	25.69	[13.10, 38.27]	32.83	[20.03, 45.64]
Population aged 65+ years (%)	15.15	[8.18, 22.13]	27.65	[20.33, 34.98]	38	[22.93, 53.08]
Years to/since intervention	38.45	[28.63, 48.28]	48.22	[40.41, 56.04]	45.26	[35.75, 54.76]
Period [post-intervention = 1; pre-intervention = 0]	−33.64	[− 107.54, 40.26]	14.09	[−50.55, 78.73]	42.19	[−23.36, 107.74]
Group [intervention = 1; control = 0]	−14.51	[− 136.30, 107.28]	−51.92	[− 165.19, 61.35]	77.44	[− 39.15, 194.03]
DiD estimator: Period * Group	16.62	[−89.90, 123.13]	−184.25	[− 300.80, − 67.70]	−199.76	[−304.04, − 95.48]

Models include random intercept for neighbourhood.

Model 1 based on 19 intervention and 95 control neighbourhoods, 1482 observations.

Model 2 based on 37 intervention and 185 control neighbourhoods, 2886 observations.

Model 3 based on 52 intervention and 260 control neighbourhoods, 4056 observations.

*CI* confidence interval; *DiD* Difference-in-Differences

### Robustness tests

During the pre-intervention period, there was no statistically significant difference in trends in emergency admission rates for respiratory conditions between the intervention and control neighbourhoods (Supplementary file, Appendix 5), suggesting that the parallel trend assumption was not violated in this analysis. We also found that including the Liverpool city-wide AQMA produced similar results.

## Discussion

This study evaluated the health impact of local air quality management policies, and explored differential effects by socioeconomic deprivation, using natural experimental methods. We found that declaring an AQMA was associated with a reduction in emergency admissions for respiratory conditions. Plausible explanations for this are that once an AQMA has been declared, an action plan is developed and measures are taken to improve air quality in that area, which in turn has beneficial effects for respiratory health. Our results also suggest that there are greater health gains when declaring an AQMA in more deprived communities.

### Strengths and limitations

This study had a number of strengths. To the best of our knowledge, this is the first study to evaluate the impact of declaring an AQMA on health and health inequalities using robust quasi-experimental methods. Applying a combination of propensity score matching and difference-in-differences, we were able to evaluate the effect of declaring an AQMA in real-world settings, and providing the parallel trend assumption was met – our analysis would have effectively controlled for all time-invariant differences between the intervention and control neighbourhoods, as well as time-varying factors that affect the outcome in the same way between the neighbourhoods.

Furthermore, by focusing directly on health outcomes, our study demonstrates a novel strategy that can be used to evaluate other air quality policies and interventions, reducing the need for air pollution exposure estimates which can be prone to measurement error and resultant bias in epidemiological studies [34]. Due to uncertainties around modelled air pollution exposure estimates, which are not produced at LSOA level, we did not include these estimates in our models. However, by averaging Defra’s modelled air pollution estimates we show in the Supplementary file that the intervention neighbourhoods experienced a greater percentage change decrease in air pollution estimates pre-post AQMA declaration compared to the control neighbourhoods. Whilst only eight of the AQMAs included in the analysis have been revoked, it should be noted that local authorities are not legally obliged to revoke AQMAs once air quality objectives are met, and may keep orders in place to prevent future degradation of air quality [16].

A limitation of the analysis is that we were only able to assess the impact of declaring an AQMA. Our results may therefore reflect the impact of measures that were taken to improve air quality, or simply an increased awareness of local air quality issues arising from the action planning process. Previous attempts to evaluate the benefits of action plan measures on air quality have been hindered by the lack of quantifiable objectives, in terms of the expected emissions or concentration reductions, provided within the plans [18]. For this study we performed content analyses of action plans from the North West Coast region (Supplementary file, Appendix 1), however it was not possible to accurately determine if, when and to what extent the proposed measures were implemented, which precluded further analysis. This may be an avenue for future qualitative or bibliographic research.

In terms of study design, methodological limitations of ecological studies can include ecological bias whereby associations present at the group-level are not apparent at the individual-level, possibly due to unmeasured confounding or measurement error [35]. Data were aggregated to relatively small areas (neighbourhoods containing 1000 to 3000 people) which likely limited the effects of ecological bias, however it should be noted that the results reflect the population-level impact of declaring an AQMA. Individuals may experience additional exposures outwith their area of residence which may increase their risk of hospitalisation for respiratory conditions, however it seems unlikely that systematic differences in these exposures between those living in AQMA and control neighbourhoods could explain our results.

### Implications for policy

Despite 20+ years of implementing the LAQM system, the UK has persistently been in breach of European Union limit values for NO_2_, leaving the system open to criticism that it has failed to produce any significant improvements in air quality. Some have argued, however, that local authorities have little control over national air quality and transport policies which have also been heavily criticised [14, 17] Additionally, alongside significant cuts to local government funding in general, resources with which to implement local measures (in the form of Defra’s Air Quality Grant) have been cut by 75% in recent years, effectively undermining the ability of local authorities to introduce effective action plans [17].

A major criticism expressed of the LAQM system is that the role of local authority public health departments has been inadequately defined. Their limited involvement in the development and delivery of action plans may limit effectiveness [20]. Despite the obvious implications for human health, air quality policy has largely been considered an environmental issue [17]. This is reflected in all processes of the LAQM system, from review and assessment, to action planning and evaluation – there are no explicit requirements to measure health or social impacts [20, 36].

Our research suggests that the LAQM system has great potential to improve health, especially amongst more socioeconomically deprived communities. These findings highlight the importance of previous calls to develop an LAQM system with a greater focus on public health, and that the effectiveness of air quality management can be enhanced by linking it with wider public health interventions and policies [20, 37]. Importantly, better integration of local health data within risk assessment and surveillance processes is needed to inform action plan measures that are based on health needs and to enable thorough evaluation to maximise health gains [20]. Whilst this may require additional resources and expertise to implement, our study demonstrates that important insights can be gleaned by directly examining the impact of air quality strategies on health outcomes.

In general, strategies to reduce air pollution are likely to have a positive impact on reducing health inequalities because air pollution concentrations tend to be highest in deprived areas. Furthermore, previous studies have found that deprivation strengthens associations between air pollution and mortality, suggesting that deprivation amplifies the negative effects of air pollution on health [37]. Adding to this evidence base, our findings suggest that health gains are greatest when air quality measures are implemented in more deprived communities, and thus the LAQM system may present a strategy to reduce inequalities in health.

Air quality strategies which address the social determinants of health (i.e. the conditions in which we are born, grow, work, live, and age [38]) have great potential to provide long-term health benefits. Examples may include infrastructure improvements to enable and promote active travel, introducing greener urban landscapes, reducing fuel poverty via investment in heating and insulation programmes, reducing traffic density and noise, and improving road safety. Introducing lasting positive improvements to the built environments of deprived communities and measuring the impacts of different measures on health inequalities should be considered priority objectives of the LAQM system.

## Conclusions

Framing air quality policy as predominantly an environmental concern has meant that considerations of the impact of the LAQM system on health have been neglected. Our results suggest that in the North West Coast region the LAQM system has contributed to a reduction in emergency hospital admissions for respiratory conditions, and may represent an effective strategy to reduce inequalities in health. These findings highlight the importance of measuring the success of air quality policies not just in terms of air pollution but also in terms of population health metrics. Public health departments should have meaningful involvement in the development and delivery of the LAQM system in order to maximise its potential to protect and improve health and reduce inequalities. Importantly, local authorities must be adequately resourced and supported to deliver effective solutions, especially in the most deprived communities where the introduction of air quality measures are likely to produce the greatest health gains.

## Supplementary Information

**Additional file 1: Appendix 1.** Air Quality Action Plans. **Appendix 2.** Air Quality Management Areas in the North West Coast. **Appendix 3.** Outline of the Difference-in-Differences analysis. **Appendix 4.** Measures and data sources. **Appendix 5.** Robustness tests.

## Data Availability

The Hospital Episode Statistics data that support the findings of this study are available from NHS Digital but restrictions apply to the availability of these data, which were used under license for the current study, and so are not publicly available. The publically available datasets generated and/or analysed during the current study have been cited within the manuscript and are available from the ONS, Place-based Longitudinal Data Resource, Ministry of Housing, Communities & Local Government, and Consumer Data Research Centre [26–32].

## Abbreviations

AQMA: Air Quality Management Area
COPD: Chronic Obstructive Pulmonary Disease
Defra: Department for Environment, Food and Rural Affairs
LAQM: Local Air Quality Management
LSOA: Lower-layer Super Output Area
NHS: National Health Service
NO_2_: Nitrogen dioxide
ONS: Office for National Statistics
