# Supplementary material for: Impact of local air quality management policies on emergency hospitalisations for respiratory conditions in the North West Coast region of England: a longitudinal controlled ecological study
Source: Int J Equity Health. 2021 Dec 13;20:254. doi: 10.1186/s12939-021-01598-w (PMC8670133; doi:10.1186/s12939-021-01598-w)
Supplement: Supplementary file 1 — Additional file 1: Appendix 1. Air Quality Action Plans. Appendix 2. Air Quality Management Areas in the North West Coast. Appendix 3. Outline of the Difference-in-Differences analysis. Appendix 4. Measures and data sources. Appendix 5. Robustness tests. [file 12939_2021_1598_MOESM1_ESM.pdf]

Supplementary file

Appendix 1: Air Quality Action Plans

Interventions/measures described within Air Quality Action Plans (AQAPs) (that were available online) in the North West Coast region have been summarised in the table and figure below. Several recurring themes were identified amongst the interventions/measures. The most commonly occurring theme was traffic management. Inclusion of interventions/measures in the action plans does not necessarily mean that they were implemented.

Table: Themes identified within AQAPs in the North West Coast region

Theme	Examples of interventions/measures
Traffic management	To reduce congestion and improve traffic flow via signage, signalling, rerouting/relocating, scheduling commercial delivery times/HGV booking systems at ports, enforcement/restrictions on obstructive parking, surveying, chicanes, height/weight restrictions, speed restrictions, junction efficiency and/or bus stop relocation
Private vehicle emissions	To reduce emissions via roadside emissions testing, anti-idling legislation (fixed penalty notices to drivers of idling vehicles), increasing parking charges, restrict parking permissions, car clubs/sharing, increase uptake of shopping deliveries, increasing toll charges and/or CAZs to incentivise use of cleaner vehicles
Public transport emissions	To reduce emissions via roadside emissions testing, procuring sustainable fleets, retrofitting buses with NO_2 abatement technology, working with bus operators to promote use of low emission buses, bus quality contracts when renewing bus contracts and/or CAZs to incentivise use of cleaner vehicles
Commercial vehicle emissions	To reduce emissions via roadside emissions testing, MOT-type tests for taxis/private hire vehicles, visual assessment of idling taxis, licensing & scrappage schemes to incentivise use of cleaner vehicles, survey commercial route users & engage local businesses, CAZs to incentivise use of cleaner vehicles, vehicle accreditation schemes, anti-idling legislation, increasing toll charges and/or Taxis Quality Partnership
Development planning	To ensure new residential development proposals mitigate potential air quality impacts, which may involve consultation with Environmental Protection, maximising use of sustainable modes of transport, section 106 agreements, guiding use of Community Infrastructure Levy, air quality assessments for major developments, assess & minimise dust & machinery emissions from demolition and construction, green space/infrastructure, Home Zones and/or planning conditions incorporating Low Emissions Strategy measures

Green infrastructure	To mitigate pollution by 'greening-up' public infrastructure via green walls, green roofs, tree planting and/or Air Quality Barriers
Cycling/walking infrastructure	To increase uptake of active travel via provision of secure cycle storage, cycle lanes, pathways, integrated travel passes, cycle hire schemes and/or advance stop lines
Public awareness/ information	To provide information regarding local air quality, to promote public transport and active travel, and encourage use of cleaner fuels/vehicles via electronic display boards, websites, hotlines, community involvement and/or cycling/walking events
Public transport routes/improvements	To increase use of public transport via improvements to bus stops/train stations, signage, maintaining/increasing subsidies, bus lanes, route/scheduling improvements, Bus Services Act, Transport Hubs at local rail stations, Park and Ride facilities, increase parking near rail/bus services, incentivised tickets and/or improve local rail services
Electric vehicle (EV) charging	To increase uptake of low emission vehicles by developing EV charging infrastructure via residential/commercial development planning, town planning and/or council car parks & workplaces
Travel plans	To encourage workplaces and schools to design, deliver and maintain travel plans which are packages of site-specific actions designed to encourage safe, healthy and sustainable travel options
Industrial emissions	To reduce emissions via environmental permits/ regulatory control and monitoring
Domestic emissions	To reduce emissions via smoke control areas, energy retrofit programme & boiler scrappage scheme and/or bonfire emissions
Lobbying/engaging central government	To encourage action by central government to improve air quality via provision of funding, national regulations, legislation and guidance, financial incentives and/or co-ordinating air quality actions across departments
Monitoring/modelling improvements	To improve air quality monitoring via provision of diffusion tubes, reviewing provision and locations and/or developing nuanced local models

Figure: Content analysis of AQAPs in the North West Coast region

	Traffic management	Private vehicle emissions	Public transport emissions	Commercial vehicle emissions	Planning	Green infrastructure	Cycling/walking infrastructure	Public awareness/information	Public transport routes/improvements	Electric vehicle charging	Travel plans	Industrial emissions	Domestic emissions	Lobbying/engaging central government	Monitoring/modelling improvements	Other
**Cheshire West and Chester**																
AQAP Whitby Rd./Station Rd., 2007	■	■	■	■	■		■	■	■		■				■	
AQAP Thornton le Moors, 2017								■				■			■	
AQAP Frodsham, 2018	■		■	■				■		■	■				■	
**Liverpool City Region**																
AQAP, 2007	■	■	■	■	■		■	■	■		■			■		Community travel support services
AQAP Combined Authority, 2019	■	■	■	■	■	■	■	■	■	■			■	■	■	Co-ordinate local strategies
**Sefton** AQAP for AQMAs 1-5, 2015	■			■	■	■	■	■			■	■				Port expansion mitigation
**Blackburn w Darwen** AQAP, 2007	■	■	■		■		■		■		■	■	■			Mosque marshalling
**Lancaster City Council**																
AQAP Lancaster, 2007	■	■			■		■		■		■					
AQAP Carnforth, 2010	■	■	■								■					
**Cheshire East** AQAP, 2011	■	■	■	■	■	■	■	■	■		■			■	■	Ensure sat nav companies have updated maps
**Carlisle** AQAP, 2012	■	■			■	■	■	■	■		■	■	■			
**St Helens** AQAP, 2013	■	■	■	■	■	■		■								
**Wyre** AQAP, 2011	■		■	■	■		■		■		■				■	
**West Lancashire** AQAP, 2011	■	■	■				■				■					
**Halton** AQAP 2014-2019	■		■		■		■				■					
**Rossendale** AQAP, 2016	■	■									■					
**Warrington**																
Air Quality Management Plan, 2003	■	■	■	■	■		■	■	■		■	■	■	■	■	
AQAP 2017-2022	■	■	■	■	■	■	■	■	■	■	■		■	■	■	
**Preston**																
AQAP, 2009	■	■					■		■		■				■	Actions in Preston's Climate Change Action Plan
AQAP, 2014	■							■	■							

Appendix 2: Air Quality Management Areas in the North West Coast

We analysed data from the North West Coast region of England which covers 29 local authorities. In total, 78 AQMAs have been declared in the North West Coast, of which 48 were declared within our intervention time frame between 2006 and 2016.

**Air Quality Management Areas in North West Coast region of England**							
**Data extracted from: https://uk-air.defra.gov.uk/aqma/list**							
**Local Authority**	**AQMA Name**	**Pollutants**	**Date Declared**	**Date Amended 1st**	**Date Amended 2nd**	**Date Revoked**	**Study intervention time frame**
South Lakeland	Kendal AQMA	Nitrogen dioxide NO_2	05/05/2001	23/11/2010			No
Warrington	Warrington AQMA No.1	Nitrogen dioxide NO_2	01/11/2001				No
Lancaster	City of Lancaster AQMA	Nitrogen dioxide NO_2	12/03/2004	01/04/2017			No
Cheshire East	Congleton AQMA No.1 Cranage	Nitrogen dioxide NO_2	01/05/2005	30/11/2006		01/06/2018	No
Cheshire East	Congleton AQMA No.2 West Road, Congleton	Nitrogen dioxide NO_2	01/05/2005	30/11/2006			No
Cheshire East	Congleton AQMA No.4 The A34 And A54	Nitrogen dioxide NO_2	01/05/2005	30/11/2006			No
Cheshire East	Congleton AQMA No3	Nitrogen dioxide NO_2	01/05/2005			30/11/2006	No
Cheshire West and Chester	Whitby Rd/Station Rd AQMA	Nitrogen dioxide NO_2	16/05/2005				No
Blackpool	Blackpool AQMA	Nitrogen dioxide NO_2	01/07/2005				No
South Ribble	AQMA 3 Lostock Hall	Nitrogen dioxide NO_2	15/08/2005				No
South Ribble	AQMA 4 - Bamber Bridge	Nitrogen dioxide NO_2	15/08/2005				No
Preston	AQMA No. 1	Nitrogen dioxide NO_2	01/09/2005				No
Preston	AQMA No.2	Nitrogen dioxide NO_2	01/09/2005				No
South Ribble	AQMA No.1	Nitrogen dioxide NO_2	01/09/2005				No
South Ribble	AQMA No.2	Nitrogen dioxide NO_2	01/09/2005				No
Blackburn with Darwen	AQMA 1 - Intack	Nitrogen dioxide NO_2	18/10/2005				No
Blackburn with Darwen	AQMA 2 - Bastwell	Nitrogen dioxide NO_2	18/10/2005				No

Blackburn with Darwen	AQMA 3 - A666 between Robert Street and Wraith Street	Nitrogen dioxide NO_2	18/10/2005			27/09/2019	No
Blackburn with Darwen	AQMA 4 - Witton	Nitrogen dioxide NO_2	18/10/2005			27/09/2019	No
Blackburn with Darwen	AQMA 5 - Earcroft	Nitrogen dioxide NO_2	18/10/2005			27/09/2019	No
Carlisle	A7 AQMA	Nitrogen dioxide NO_2	02/12/2005	25/07/2019			No
Warrington	Warrington AQMA No.2	Nitrogen dioxide NO_2	01/02/2006			30/11/2016	Yes
Cheshire East	Nantwich AQMA	Nitrogen dioxide NO_2	15/12/2006				Yes
Carlisle	AQMA No.2	Nitrogen dioxide NO_2	26/01/2007				Yes
Lancaster	Carnforth AQMA	Nitrogen dioxide NO_2	10/04/2007				Yes
Burnley	Duke Bar AQMA	Nitrogen dioxide NO_2	12/10/2007			24/01/2011	Yes
Cheshire West and Chester	AQMA Boughton No2	Nitrogen dioxide NO_2	01/03/2008	11/08/2011		23/05/2017	Yes
Cheshire East	Congleton AQMA No.5 (Lower Heath)	Nitrogen dioxide NO_2	01/04/2008				Yes
Cheshire East	Congleton AQMA No.6 (Sandbach)	Nitrogen dioxide NO_2	01/04/2008				Yes
Cheshire East	Chester Road AQMA	Nitrogen dioxide NO_2	24/04/2008				Yes
Cheshire East	Nantwich Road AQMA Crewe	Nitrogen dioxide NO_2	24/04/2008	01/05/2012			Yes
Carlisle	AQMA No.3	Nitrogen dioxide NO_2	01/08/2008			03/07/2019	Yes
Carlisle	AQMA No.4	Nitrogen dioxide NO_2	01/08/2008				Yes
Carlisle	AQMA No.5	Nitrogen dioxide NO_2	01/08/2008				Yes
Carlisle	AQMA No.6	Nitrogen dioxide NO_2	01/08/2008			03/07/2019	Yes
Sefton	AQMA 1	Particulate Matter PM_{10}	15/01/2009			01/08/2016	Yes
Sefton	AQMA 2	Nitrogen dioxide NO_2	15/01/2009	07/12/2015	01/08/2016		Yes
Sefton	AQMA 3	Nitrogen dioxide NO_2 and particulate matter PM_{10}	15/01/2009	07/12/2015	01/08/2016		Yes
Liverpool	Liverpool City AQMA	Nitrogen dioxide NO_2	01/04/2009				Yes
St. Helens	M6 AQMA No.1	Nitrogen dioxide NO_2	30/04/2009				Yes
St. Helens	Newton High Street AQMA (No.2)	Nitrogen dioxide NO_2	30/04/2009				Yes
Wyre	Chapel	Nitrogen dioxide NO_2	01/08/2009				Yes

	Street AQMA						
Lancaster	Galgate AQMA	Nitrogen dioxide NO_2	16/11/2009				Yes
West Lancashire	Ormskirk AQMA	Nitrogen dioxide NO_2	20/01/2010				Yes
Cheshire East	Earl Street Crewe	Nitrogen dioxide NO_2	31/01/2010	01/04/2012			Yes
Cheshire East	Disley AQMA	Nitrogen dioxide NO_2	01/04/2010				Yes
Cheshire East	Knutsford AQMA	Nitrogen dioxide NO_2	01/04/2010				Yes
Cheshire East	Macclesfield AQMA	Nitrogen dioxide NO_2	01/04/2010				Yes
Ribble Valley	Whalley Road, Clitheroe No 1	Nitrogen dioxide NO_2	31/05/2010				Yes
Warrington	Warrington Borough Council AQMA Order no 3 2010	Nitrogen dioxide NO_2	04/06/2010			30/11/2016	Yes
Halton	Halton AQMA No 2	Nitrogen dioxide NO_2	01/03/2011				Yes
Halton	Halton Widnes No 1	Nitrogen dioxide NO_2	01/03/2011				Yes
Pendle	Air Quality Management Area Colne	Nitrogen dioxide NO_2	01/04/2011				Yes
Cheshire East	Wistaston Road Crewe AQMA	Nitrogen dioxide NO_2	01/11/2011				Yes
St. Helens	AQMA No. 3 Borough Rd	Nitrogen dioxide NO_2	30/11/2011				Yes
St. Helens	AQMA No.4 (Reflection Court)	Nitrogen dioxide NO_2	30/11/2011				Yes
Sefton	AQMA 4	Nitrogen dioxide NO_2	01/02/2012	07/12/2015	01/08/2016		Yes
Sefton	AQMA 5	Nitrogen dioxide NO_2	01/02/2012	07/12/2015	01/08/2016		Yes
Blackburn with Darwen	AQMA No 6 Blackamoor	Nitrogen dioxide NO_2	08/02/2012				Yes
Blackburn with Darwen	AQMA No 7 Four Lane Ends	Nitrogen dioxide NO_2	08/02/2012				Yes
Blackburn with Darwen	AQMA No 8 Accrington Road and Burnley Road	Nitrogen dioxide NO_2	08/02/2012			22/12/2017	Yes
Preston	AQMA No. 3	Nitrogen dioxide NO_2	01/05/2012				Yes
Preston	AQMA No. 4	Nitrogen dioxide NO_2	01/05/2012				Yes
Rossendale	Rossendale AQMA 1	Nitrogen dioxide NO_2	08/01/2013				Yes
Rossendale	Rossendale AQMA 2	Nitrogen dioxide NO_2	08/01/2013				Yes
Preston	AQMA No. 5	Nitrogen dioxide NO_2	01/03/2014				Yes

Cheshire West and Chester	Frodsham AQMA Cheshire West and Chester	Nitrogen dioxide NO_2	27/11/2015				Yes
Cheshire West and Chester	Thornton le Moors AQMA No. 4	Sulphur dioxide SO_2	30/09/2016				Yes
Warrington	Warrington AQMA 4 2016	Nitrogen dioxide NO_2	30/11/2016				Yes
Cheshire West and Chester	Chester City Centre AQMA (No.5)	Nitrogen dioxide NO_2	23/05/2017				No
Cheshire East	Broken Cross, Macclesfield AQMA	Nitrogen dioxide NO_2	01/10/2017				No
Cheshire East	Chester Road, Middlewich AQMA	Nitrogen dioxide NO_2	01/10/2017				No
Cheshire East	Hibel Road, Macclesfield AQMA	Nitrogen dioxide NO_2	01/10/2017				No
Cheshire East	Middlewich Road, Sandbach AQMA	Nitrogen dioxide NO_2	01/10/2017				No
Cheshire East	Park Lane, Macclesfield AQMA	Nitrogen dioxide NO_2	01/10/2017				No
South Ribble	AQMA Order 5 Leyland	Nitrogen dioxide NO_2	01/01/2018				No
Cheshire East	Chelford Road, Knutsford	Nitrogen dioxide NO_2	01/11/2019				No
Cheshire East	Lewin Street, Middlewich	Nitrogen dioxide NO_2	01/11/2019				No
Allerdale	N/A	N/A	N/A				N/A
Barrow-in-Furness	N/A	N/A	N/A				N/A
Chorley	N/A	N/A	N/A				N/A
Copeland	N/A	N/A	N/A				N/A
Eden	N/A	N/A	N/A				N/A
Fylde	N/A	N/A	N/A				N/A
Hyndburn	N/A	N/A	N/A				N/A
Knowsley	N/A	N/A	N/A				N/A
Wirral	N/A	N/A	N/A				N/A

Maps showing intervention neighbourhoods and matched control neighbourhoods located within the North West Coast region of England, by deprivation subgroup

Least deprived subgroup

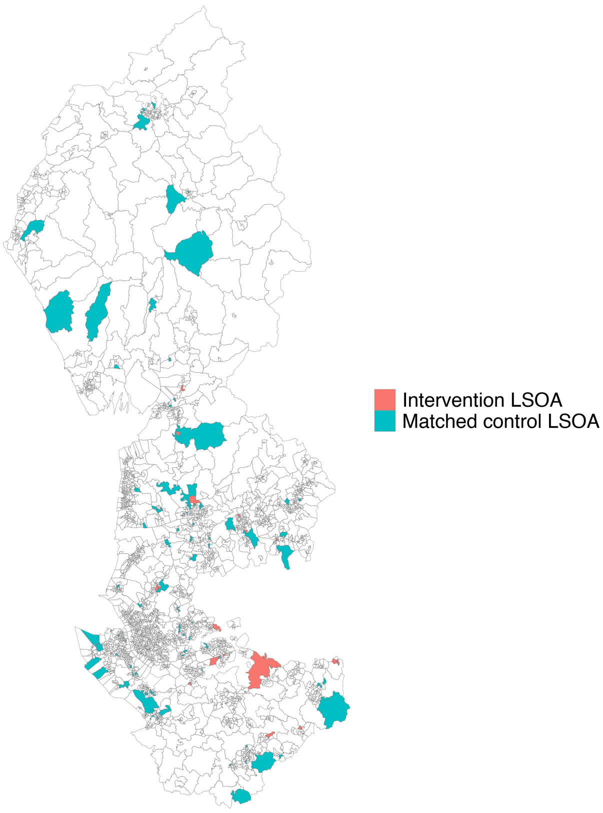

Middle

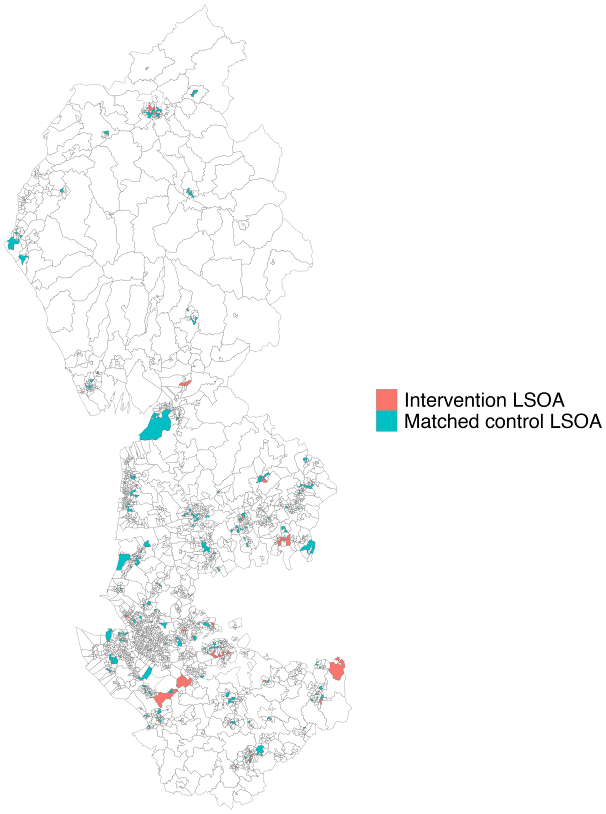

Most deprived subgroup

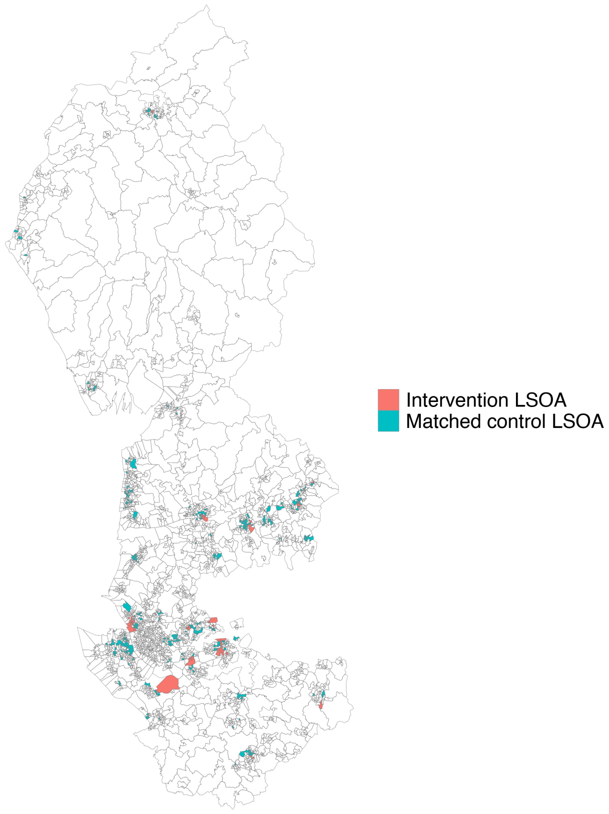

Appendix 3: Outline of the Difference-in-Differences analysis

Difference-in-differences (DiD) analyses are an established approach used in econometrics[1, 2] and increasingly in health research[3, 4] for evaluating the impact of interventions, where the researcher has not manipulated the assignment of the intervention, sometimes known as "natural experiments".

In DiD analyses, outcomes are observed for two groups before and after an intervention and one of the groups is exposed to a treatment in the second period but not in the first period, and the second group is not exposed to the treatment during the full time frame. The average change in outcomes in the second (control) group is then subtracted from the average change in outcomes the first (treatment) group. This removes biases in second period comparisons between the treatment and control group that could be the result from permanent differences between those groups, as well as biases from comparisons over time in the treatment group that could be the result of trends.

Thus the differences-in-differences estimator is therefore:

$$\hat{\gamma} = \left(\bar{Y}_{Treatment,AFTER} - \bar{Y}_{Treatment,BEFORE}\right) - \left(\bar{Y}_{COMPARATOR,AFTER} - \bar{Y}_{COMPARATOR,BEFORE}\right)$$

$\bar{Y}$ is the mean of the outcome variable in the intervention areas after the start of the intervention ($\bar{Y}_{Treatment,AFTER}$) and before the start of the intervention ($\bar{Y}_{Treatment,BEFORE}$) and in the comparator areas after the start of the intervention ($\bar{Y}_{COMPARATOR,AFTER}$) and before the start of the intervention ($\bar{Y}_{COMPARATOR,BEFORE}$). A representation of this is given in the figure below:

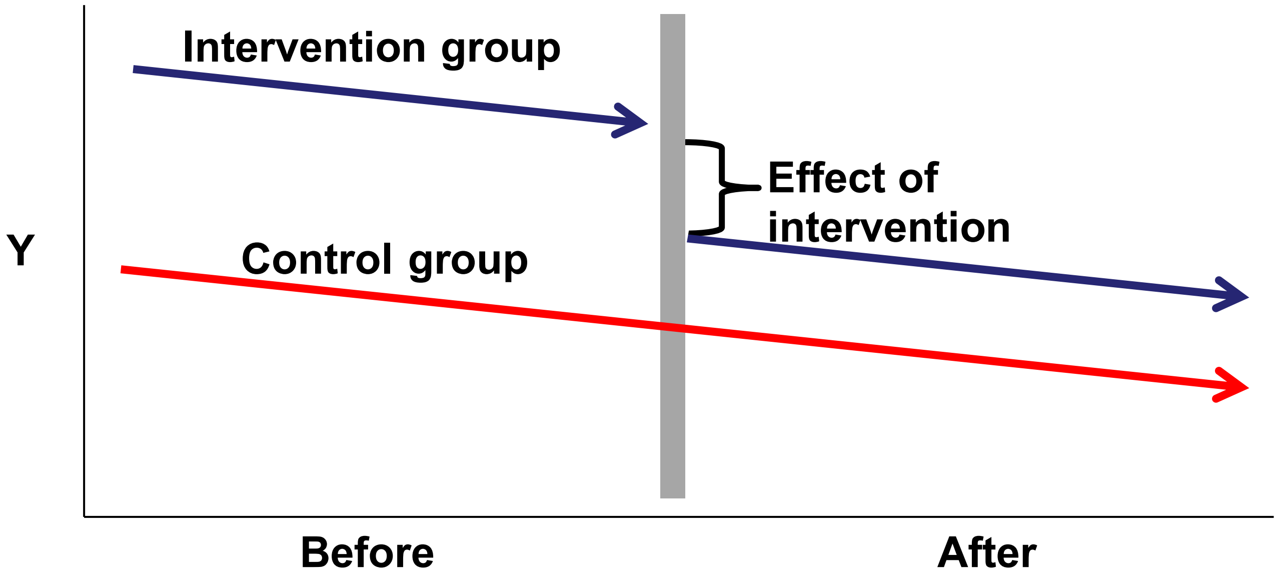

Whilst this analysis cannot be biased due to time invariant differences between the intervention and control groups, it could be biased by trends in other predictors of the outcome if these diverged between treatment and intervention groups after the intervention. We therefore additionally include potential time varying confounders in the regression model, giving the following model used in the analysis:

$$Y_{ij} = \beta_{0j} + \beta_{1j}t_{ij} + \beta_{2j}Age15_{ij} + \beta_{3j}Age65_{ij} + \beta_{4j}Unemployment_{ij} + \beta_{5j}Group_{ij} + \beta_{6j}Period_{ij} + \beta_{7j}Group_{ij}*Period_{ij} + \varepsilon_{ij}$$

Where Y_{ij} is the emergency admission rate for respiratory conditions per 100,000 population for LSOA *i* at year *j.* The intercept β_{0j} contains a random effect term and thus varies across years. *t* is a time-trend term indicating years to/since intervention. *Age15* is the percent of the population aged <15 years. *Age65* is the percent of the population aged 65+ years. *Unemployment* is the percent of the working age population (aged 16–64 years) claiming Jobseeker's Allowance or Universal Credit. *Group* indicates whether LSOA *i* is an intervention or control neighbourhood (intervention = 1; control = 0). *Period* indicates whether year *j* occurs post or pre-intervention (post-intervention = 1; pre-intervention = 0). *Group*Period* is the difference-in-differences estimator.

Appendix 4: Measures and data sources

Measure	Details
Population size and age profile of the population	Annual data on the size of the population for all ages, those aged <15 years and 65+ years per Lower Super Output Area (LSOA) were derived from mid-year population estimates provided by the Office for National Statistics (ONS) (https://www.ons.gov.uk/peoplepopulationandcommunity/populationandmigration/populationestimates/datasets/lowersuperoutputareamidyearpopulationestimates).
Unemployment	Unemployment prevalence per year was calculated using claimant data provided by the ONS. Unemployment was measured as the percentage of people aged 16–64 years claiming Jobseeker's Allowance or Universal Credit principally for the reason of being unemployed (https://pldr.org/dataset/2z6yv).
Emergency hospital admissions for respiratory conditions per 100,000 population	Emergency hospital admissions for respiratory conditions were defined using ICD-10 codes: J00-J06, J09-J18, J20-J22 and J40-J47. Annual emergency admission rates per 100,000 population were calculated using Hospital Episode Statistics (HES), with population data obtained from the ONS. Continuous inpatient (CIP) spells were used to calculate emergency admissions per calendar year.
Income deprivation score	The income score domain of the Index of Multiple Deprivation 2015 data were provided by the Ministry of Housing, Communities & Local Government at LSOA level (https://www.gov.uk/government/statistics/english-indices-of-deprivation-2015).
Prevalence of asthma and chronic obstructive pulmonary disease (COPD)	Quality and Outcomes Framework (QOF) indicators for the prevalence of asthma, and COPD. Weighted averages of QOF indicators per LSOA were calculated using data provided by NHS Digital on the number of patients registered per general practice per LSOA (https://pldr.org/dataset/e6nzv ; https://pldr.org/dataset/23q1e).
Urban/rural classification	The Rural Urban Classification (2011) was obtained from the ONS at LSOA level (https://data.gov.uk/dataset/b1165cea-2655-4cf7-bf22-dfbd3cdeb242/rural-urban-classification-2011-of-lower-layer-super-output-areas-in-england-and-wales).
Distance to the nearest general practice and hospital	The Consumer Data Research Centre provided data per LSOA on the average road network distance to the nearest hospital with an Accident and Emergency (A&E) department, and the nearest general practice. Road network distances in kilometres were calculated by deriving the fastest route by car to travel from each postcode within an LSOA to the nearest health service (https://data.cdrc.ac.uk/dataset/access-to-healthy-assets-and-hazards-ahah).

Appendix 5: Robustness tests

Parallel trends

We investigated the parallel trends assumption by comparing trends in the outcome of interest between the intervention and control neighbourhoods in the time period before AQMA declaration, using the following regression model:

$$Y_{ij} = \beta_{0j} + \beta_{1j}Unemployment_{ij} + \beta_{2j}Age15_{ij} + \beta_{3j}Age65_{ij} + \beta_{4j}t_{ij} + \beta_{5j}Group_{ij} + \beta_{6j}t_{ij}*Group_{ij} + \varepsilon_{ij}$$

Where Y_{ij} is the emergency admission rate for respiratory conditions per 100,000 population for LSOA *i* at year *j* (within the pre-intervention period). The intercept β_{0j} contains a random effect term and thus varies across years. *Unemployment* is the percent of the working age population (aged 16–64 years) claiming Jobseeker's Allowance or Universal Credit. *Age15* is the percent of the population aged <15 years. *Age65* is the percent of the population aged 65+ years. *t* is a time-trend term indicating years to AQMA declaration. *Group* indicates whether LSOA *i* is an intervention or control neighbourhood (intervention = 1; control = 0).

The *t*Group* estimator below indicates that there was no statistically significant difference in trends in emergency admission rates for respiratory conditions between the intervention and control neighbourhoods in the time period before AQMA declaration, suggesting that the parallel trend assumption was not violated in this analysis.

Analysis showing trends in emergency admissions for respiratory conditions per 100,000 population in the intervention relative to the control neighbourhoods in the pre-intervention period

	Coefficient	SE	95% CI	t-value	p-value
Working age population unemployed (%)	34.81	6.55	[21.97, 47.64]	7.8	< .001
Population aged <15 years (%)	47.45	4.49	[38.65, 56.24]	12.08	< .001
Population aged 65+ years (%)	19.63	3.43	[12.91, 26.36]	6.5	< .001
Years to intervention	25.4	3.72	[18.10, 32.69]	9.79	< .001
Group [intervention = 1; control = 0]	76.66	48.54	[-18.49, 171.81]	1.5	0.114
Years to intervention * Group	0.55	7.89	[-14.92, 16.03]	0.09	0.944

Model includes random intercept for LSOA
Model based on 108 intervention and 540 control LSOAs, and 4566 observations
CI = confidence interval; LSOA = Lower-layer Super Output Area

Analysis showing trends in emergency admissions for respiratory conditions per 100,000 population in the intervention relative to the control neighbourhoods in the pre-intervention period, stratified by income deprivation

	Model 1: Least deprived neighbourhoods		Model 2: Middle deprivation		Model 3: Most deprived neighbourhoods	
	Coefficient	95% CI	Coefficient	95% CI	Coefficient	95% CI
Working age population unemployed (%)	-3.48	[-49.01, 42.06]	-18.28	[-43.71, 7.14]	15.7	[0.16, 31.24]
Population aged <15 years (%)	16.23	[1.42, 31.04]	22.67	[11.45, 33.89]	30.04	[17.50, 42.58]
Population aged 65+ years (%)	16.25	[8.76, 23.74]	23.26	[16.04, 30.48]	36.44	[19.92, 52.96]
Years to intervention	31.61	[19.49, 43.74]	45.25	[34.34, 56.15]	16.14	[3.88, 28.40]
Group [intervention = 1; control = 0]	19.71	[-139.40, 178.82]	-63.37	[-187.47, 60.73]	60.38	[-67.66, 188.42]
Years to intervention * Group	10.76	[-16.77, 38.29]	0.89	[-22.11, 23.89]	0.29	[-23.29, 23.87]

Models include random intercept for neighbourhood
Model 1 based on 19 intervention and 95 control neighbourhoods, 798 observations
Model 2 based on 37 intervention and 185 control neighbourhoods, 1572 observations
Model 3 based on 52 intervention and 260 control neighbourhoods, 2196 observations
CI = confidence interval

Including Liverpool city-wide AQMA

Results of difference-in-differences analysis showing the change in emergency admissions for respiratory conditions per 100,000 population in the intervention neighbourhoods following the declaration of an AQMA relative to the control neighbourhoods, including neighbourhoods in Liverpool

	Coefficient	95% CI	p-value
Working age population unemployed (%)	11.98	[6.97, 17.00]	< .001
Population aged <15 years (%)	45.16	[40.75, 49.57]	< .001
Population aged 65+ years (%)	16.89	[13.63, 20.16]	< .001
Years to/since intervention	60.71	[57.59, 63.82]	< .001
Period [post-intervention = 1; pre-intervention = 0]	-105.12	[-126.90, -83.34]	< .001
Group [intervention = 1; control = 0]	393.9	[331.63, 456.17]	< .001
DiD estimator: Period * Group	-423.57	[-463.84, -383.31]	< .001

Model includes random intercept for neighbourhood
Model based on 442 intervention and 1768 control neighbourhoods, and 28,730 observations
CI = confidence interval; DiD = Difference-in-Differences

Air pollution averages in the intervention and control neighbourhoods, before and after AQMA declaration

**NO2**	Before	After	Percentage change
Intervention neighbourhoods	16.09	14.21	-11.7
Control neighbourhoods	14.25	12.78	-10.3

**SO2**	Before	After	Percentage change
Intervention neighbourhoods	3.45	2.38	-31.2
Control neighbourhoods	2.86	2.10	-26.7

**PM10**	Before	After	Percentage change
Intervention neighbourhoods	14.08	12.62	-10.4
Control neighbourhoods	13.46	12.22	-9.2

References

1. Angrist JD, Pischke J-S. Mostly Harmless Econometrics: An Empiricist's Companion. Princeton: Princeton University Press 2009.

2. Wooldridge JM. Econometric Analysis of Cross Section and Panel Data. 2nd Revised ed. Cambridge: Mass: MIT Press 2010.

3. Craig P, Dieppe P, Macintyre S, et al. Developing and evaluating complex interventions: the new Medical Research Council guidance. BMJ 2008;337:a1655. doi: 10.1136/bmj.a1655.

4. Craig P, Katikireddi SV, Leyland A, et al. Natural Experiments: An Overview of Methods, Approaches, and Contributions to Public Health Intervention Research. Annu Rev Public Health 2017;38:39-56. doi: 10.1146/annurev-publhealth-031816-044327.
